# Ozonation of Hot Red Pepper Fruits Increases Their Antioxidant Activity and Changes Some Antioxidant Contents

**DOI:** 10.3390/antiox8090356

**Published:** 2019-09-01

**Authors:** Monika Sachadyn-Król, Małgorzata Materska, Barbara Chilczuk

**Affiliations:** Department of Chemistry, University of Life Sciences in Lublin, 20-950 Lublin, Poland

**Keywords:** ozone, *Capsicum annuum*, antioxidants, phenolic compounds

## Abstract

The effect of treatment of pepper fruits with gaseous ozone and storage time following the ozonation process on changes in the content of lipophilic fraction is analyzed for the first time in this paper. The aim of the present study was to assess the impact of ozone treatment on the composition of lipophilic compound fraction and its antioxidant activity (AA). Pepper fruits of cv. Cyklon were ozonated for 1 and 3 h immediately after harvesting. Then, the fruits were stored for 30 days under refrigeration conditions. The total content of phenolic compounds and the AA of the lipophilic fraction isolated from the pericarp and placenta of the fruits were investigated after 10, 20, and 30 days of storage. Additionally, quantitative high-performance liquid chromatography diode array detection analysis of individual phenolic compounds was performed. The results revealed that the content and activity of secondary metabolites varied during storage, with the highest values recorded on the 20th day after harvest, both in control and ozonated fruits, regardless of the ozone dosage used. Treatment of the fruits with ozone for 3 h, but not for 1 h, exhibited a positive effect on the phenolic composition and AA during the prolonged storage of pepper fruits. Three hours of ozonation seems to be the appropriate time to increase the persistence of pepper fruits during storage.

## 1. Introduction

The consumption of raw fruits and vegetables has seen an increasing trend in the last few years due to growing nutritional awareness among the population [1]. In addition, consumer demands for minimally processed foods with natural flavor and taste has led to the development of a number of preservation strategies whose main focus was to extend the shelf life of fruits and vegetables [2]. Fresh or minimally processed fruits and vegetables are highly perishable and easily subjected to post-harvest microbial contaminations and physiological changes during transportation, processing, storage, and wholesale and retail trade [3]. Novel food processing and preservation technologies, while considering microbiological safety, should also ensure, at least, the preservation of ingredients at the same level, or even improvement of, the quality of the raw material [4]. Food manufacturers in association with scientists are looking for methods to extend food shelf life and improve its pro-health properties. The techniques used to achieve this goal are nanoencapsulation or electrospraying [2].

Ozone treatment is being widely used in the food industry, since this procedure involves only minimal postharvest treatments and is relatively inexpensive and available worldwide [5]. Ozone is one of the strongest oxidants exhibiting a broad spectrum of antimicrobial activity—against bacteria, fungi, viruses, protozoa, and also bacterial and fungal spores [6]. The dosage of ozone used in the industry is potent enough to disinfect the surface, which largely eliminates the problem of contamination from mold growth and microbiological infections [1]. Additionally, it also exhibits other positive properties, such as decomposition of mycotoxins and the prevention of their recurrence during the storage process [5,7], as well as the ability to degrade pesticide residues present within the food product [8,9,10]. Despite many studies being published with regard to the beneficial effects associated with the adoption of ozonation process in the food industry, there still exists mistrust among consumers [10]; therefore, the producers do not often inform about using this method. The most commonly used technique in fruit and vegetable processing is storing the raw material in an atmosphere containing low concentrations of ozone [11]. Another approach is subjecting them to a single treatment, but this method requires exposure to higher concentrations of ozone (1–5 ppm for pepper fruits). However, higher dosages of ozone and longer contact time might result in the degradation of fruit quality, owing to increased oxidative stress [12]. Thus, selection and standardization of all the appropriate parameters is of utmost importance. Hence, the ozonation technique is now being comprehensively studied. A number of studies have been conducted to confirm the positive effect of ozone treatment on the shelf life of fruits and vegetables. A few of them were specific to sweet or hot and red or green peppers [12,13,14,15,16]. Most of these studies focus on issues related to the shelf life, like disease development, affecting the microbiological quality; physiological characteristics, resulting in loss of weight, texture, and color; and physicochemical characteristics, concerning ascorbic acid content, physiological and total soluble solids content; or overall acceptability. In the literature, only the works of Glowacz [17,18] and Sachadyn-Król [19] refer to the issue of secondary metabolites in pepper in the context of ozonation.

Red pepper (*Capsicum annuum* L.) is considered to be a rich source of secondary metabolites, which include compounds with different lipophilicities. Vitamin C, phenolic acids, and their derivatives are hydrophilic compounds, while the dominating acids found in the pepper fruits are gallic and 5-*O*-caffeoylquinic acid [20]. Compounds that show medium lipophilicity are glycosidic derivatives of flavonoids, mainly quercetin and luteolin, as well as esters of ferulic and sinapic acids [21,22]. Alkaloids, capsaicin, and dihydrocapsaicin, components of hot pepper varieties and various types of capsinoids found in sweet varieties, are lipophilic compounds. The lipophilic fraction also includes terpenoids, which show varying degrees of glycosylation. They are described to be the precursors of carotenoid pigments [22]. Depending on the nature and structure of compounds present in the fruits, the impact following oxidative action of ozone on phenolic compounds may be different. These compounds can block the action of free radicals and are capable of inhibiting the reactions caused by single active atoms of oxygen. Phenolic compounds exhibit antioxidant activity (AA), which is determined by their chemical structure [23]. Previous studies [19] regarding the effect of ozone treatment on the level of secondary metabolites in stored pepper fruits revealed that ozonation for 3 h induced an increase in the concentration of compounds showing intermediate lipophilicity: quercetin 3-*O*-rhamnoside and quercetin-3-*O*-rhamnoside-7-glucoside in fruits stored for 20 days after treatment [19]. These compounds were part of the fraction isolated with 40% methanol. In the presented work, we focus on a group of lipophilic compounds that are extracted from the pepper extract using 70% methanol. Since these compounds show affinity for fat, they appear to be potent antioxidants that may show their activity at the level of the cell membrane [23]. The aim of the presented work was to evaluate the impact of ozone treatment on the composition of lipophilic compound fraction and thereby determine its AA.

## 2. Materials and Methods

### 2.1. Plant Material and Ozone Treatment

Fully matured fruits of hot pepper (*C. annuum* L. var. Cyklon) were analyzed. Plants were cultivated in a greenhouse of the Department of Cultivation and Fertilization of Horticultural Plants, University of Life Sciences, Lublin, Poland. Fully ripe and undamaged fruits (weight: 20–30 g) were selected for the investigation purpose. Fruits were divided into three groups (20 fruits in each) after harvesting, and the first group acted as the control. The other two groups were exposed to a stream of ozone gas at a concentration of 2 mg/dm^3^ for 1 h and for 3 h, respectively, at a temperature of 25 °C. All experiments were performed in triplicate. The ZY-H103 ozone generator (Zhong-Yi Electronics Co., Ltd, Fujian, China) was used. After ozone treatment, the fruits were stored in the dark at 8 °C in tightly sealed packages of LDPE film for 10 (T10), 20, (T20), and 30 (T30) days. The control fruits (without treatment) were also stored in similar conditions. For evaluation, the fruits were washed, cut, and pericarps were separated from placenta and analyzed separately. Furthermore, total phenolic content (TPC) and antiradical activity of the selected samples were estimated. In addition, the lipophilic profile of the fraction isolated from placenta and pericarp of pepper fruits was determined by the high-performance liquid chromatography (HPLC) technique.

### 2.2. Extraction and Fractionation of Polyphenols

The protocol followed for extraction and obtaining 70% methanol fraction is based on our previously published procedure [21]. The pericarp and placentae (5 g) were homogenized for 15 min in an 80% aqueous ethanol solution on a Diax 900 homogenizer (Heidolph, Schwabach, Germany). After filtration on a paper filter, the clear solution was collected and the volume was made up to 50 mL with 80% ethanol solution and mixed thoroughly. Then, 40 mL of ethanolic extract was subjected to solid-phase extraction (SPE) to enable separation of fractions with different lipophilicities. To remove alcohol from the sample, the extracts were evaporated on a rotary vacuum evaporator (Rotavapor R-100, Buchi Labortechnik AG, Flawil, Switzerland) at a temperature of 40 °C. After concentration, 5 mL of water was added and the diluted samples were applied to SepPak columns (2 g, C18; Waters, Milford, CT, USA), which were preconditioned with methanol and water. The portion of extract adsorbed on the SPE column was washed with water and 40% methanol. After washing, the fraction of lipophilic compounds was eluted using a 70% aqueous methanol solution. The obtained fractions were evaporated, transferred to tubes of 2 mL volume, and subjected to further analyses.

### 2.3. Determination of the Total Phenolic Content (TPC)

The TPC was analyzed spectrophotometrically by the Folin–Ciocalteu method [24]. Samples (60 μL) diluted with water to a volume of 0.6 mL were mixed with Na_2_CO_3_ solution (1.2 mL at a concentration of 75 g/L), and then Folin’s reagent (1.5 mL diluted with distilled water at a ratio of 1:10) was added. The prepared mixture was incubated for 30 min in the dark at room temperature and later the absorbance was measured at a wavelength of *λ* = 750 nm. Spectrophotometric analyses were done using a UV–VIS spectrophotometer (Shimadzu A-160, Shimadzu Corp., Kioto, Japan). Analyses were repeated four times, and the results were presented as means in mg of chlorogenic acid/100 g of fresh matter (FM) on the basis of calibration curve prepared for this compound.

### 2.4. DPPH Free Radical-Scavenging Assay

The assessment of antiradical activity of lipophilic fractions extracted from pepper fruits was performed by using synthetic DPPH (1,1-diphenyl-2-picrylhydrazyl) radical assay [25]. In this method, the direct reduction of the DPPH radical is indicated by a change in the color of the reaction mixture. Briefly, 0.1 mL of the analyzed sample was added to 4 mL of DPPH solution in 100% methanol (0.01 mM). In the blank sample, 0.1 mL of 70% methanol is added to 4 mL of methanol DPPH solution. After storing the samples in the shade at room temperature for 30 min, the absorbance was measured at *λ* = 515 nm with a UV–VIS spectrophotometer.

The AA, expressed as the % of DPPH radical reduction, was calculated from the formula:

(1) $$\%\mathrm{AA}=[1-\frac{As}{Ab}]\times 100\%$$

where AA is the antioxidant activity of the analyzed sample, *As* is the absorbance of the analyzed sample, and *Ab* is the absorbance of the blank sample.

### 2.5. High-Performance Liquid Chromatography

Detailed investigations regarding the changes observed in the profile of compounds present in the 70% fraction, following the ozone treatment and fruit storage, were conducted by HPLC method [21]. The analysis was done on an Empower-Pro chromatograph (Waters, Milford, CT, USA) attached to a quaternary pump (M2998; Waters, Milford, CT, USA), a degasser (In-Line Degasser AF, Waters, Milford, CT, USA), and a UV–VIS diode array detection system (2998 Photodiode Array Detector, Waters, Milford, CT, USA). A column filled with modified silica gel RP-18 (Atlantis T3, 3 μm, 4.6 mm × 150 mm; Waters, Milford, CT, USA) was used. The mobile phase consisted of A (100% acetonitrile), B (H_2_O redistilled), and C (100% methanol) solutions in a proportion in which the concentration of solvents A and C (in the same proportion) were as follows: until the 0–15th min, 18–22%; 15–27 min, 22–25%; 27–35 min, 25–30%; and 35–45 min, 35–50%. The flow speed was 1 mL/min, and the absorbance was measured at 280 nm. Quantitative analysis was based on the standard calibration curve prepared for quercetin 3-*O*-rhamnoside, and the concentration of the compounds present in 70% fraction extracted from the pericarp and placenta was expressed as equivalent of this compound (mg/100 g of FM).

### 2.6. Statistical Analysis

All analyses were carried out in four replicates, and standard deviations were calculated for each data series as an indicator of dataset scatter plots. To analyze the results obtained for the effect of time of storage, portion of fruit, and ozone treatment on the TPC and antiradical activity, a one-way analysis of variance (ANOVA) was used. The significance of the differences between the means was determined using least-squares means multiple-range test with 5% probability for error. To assess the relationship between the ozone treatment and alterations in the concentrations of compounds, a Pearson’s correlation analysis was performed. A *p*-value < 0.01 was considered to be significant. Principal component analysis (PCA) was also performed. Statistical comparisons were performed using STATGRAPHIC Centurion software, version XVI (Statgraphic Technologies, Inc., Virginia, USA).

## 3. Results and Discussion

### 3.1. TPC and Antioxidant Activity

**Figure 1:**
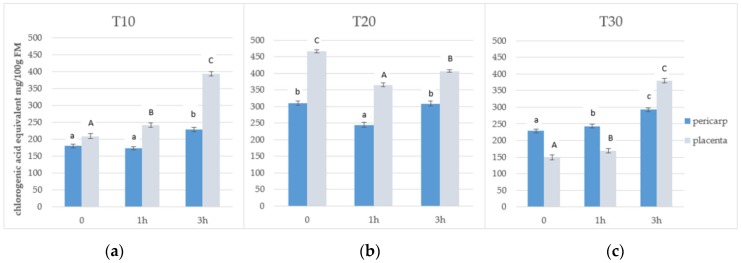
Total phenolic compounds in the 70% aqueous methanol fraction obtained from pepper fruits at three lengths of storage: (**a**) after 10 days (T10), (**b**) after 20 days (T20), and (**c**) after 30 days (T30). Notes: ^1^ Values in the graphs denoted with the same letters do not differ statistically significantly at *p* < 0.05; ^2^ Lowercase letters correspond to pericarp and uppercase letters to placenta; FM: fresh matter.

Total phenolic content and the antiradical activities of the 70% fraction were dependent on the anatomical part from where the sample was extracted, the storage time, and the ozonation process. The mean value of TPC was found to be 237.5 mg of chlorogenic acid/100 g of FM, which was much greater for placenta (358 mg) than for pericarp (245 mg). Comparable results for total phenolic compounds were previously noted in the fraction that exhibited medium lipophilicity and was eluted with 40% methanol. The mean value of TPC was found to be 229.3 mg of chlorogenic acid/100 g of FM [15]. The storage time significantly affected the average TPC values. The highest phenolic content was noted after 20 days of storage (350 mg of chlorogenic acid/100 g of FM) and the lowest at 10 days (T10) (237.5 mg of chlorogenic acid/100 g of FM). The application of ozone for 3 h caused a significant increase in the content of total phenolic compounds (Figure 1a–c). This applies to both time periods (T10 and T30). A large increase in TPC after ozonation process is particularly observed in the anatomical part of placenta. In the samples undergoing ozone treatment for 1 h, a different effect was observed. Generally, however, this exposure period is not sufficient to achieve the escalation effect.

**Figure 2:**
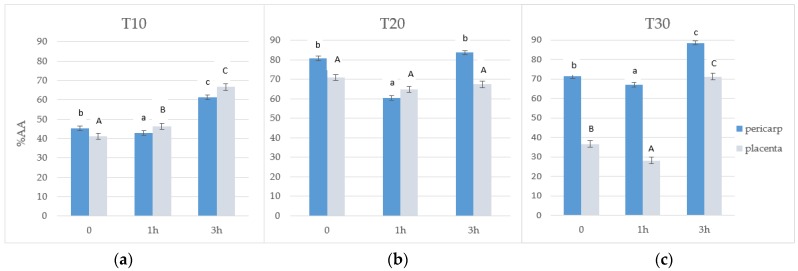
Antiradical activity (%AA) of the 70% aqueous methanol fraction obtained from pepper fruits at three lengths of storage: (**a**) after 10 days (T10), (**b**) after 20 days (T20), and (**c**) after 30 days (T30). **Notes:** ^1^ Values in the graphs denoted with the same letters do not differ statistically significantly at *p* < 0.05. ^2^ Lowercase letters correspond to pericarp and uppercase letters to placenta.

Similar results were noted for antiradical activity (AA) against DPPH, and the mean AA value was found to be 61%. The storage time had a statistically significant effect on the AA which was found to be highest after 20 days (71%) and lowest after 10 days of ozonation (51%). However, longer storage (T30) did not result in a further increase in AA (60.5%). Pericarp showed significantly higher activity compared to the placenta (67% and 55%, respectively). The ozonation period also influenced the change in the mean values of antiradical activity. Three-hour exposure to ozone gas caused an increase in the antiradical activity at 10 and 30 days of storage (Figure 2a–c). A significantly large difference can be seen for the placental sample after 30 days.

The results show higher phenolic content at T20 and T30 for placenta compared to pericarp, but the opposite trend is observed for AA, which was found to be higher for pericarp. This discrepancy may be explained based on the fact that placenta contains some phenolic compounds that possess lipophilic character but do not exhibit AA, e.g., lignin [26,27].

Our findings suggest that the content of phenolic compounds and AA are affected by ozone in a dose-dependent manner. Differences in TPC and AA of 70% aqueous methanol fraction obtained after 1 and 3 h of ozone treatment can be explained, similar to the 40% fraction [19], by the ozonation process itself and its effect on metabolism. When whole fruits were exposed to the ozone gas, the outer portion is mainly exposed to oxidative stress. The shorter ozone treatment (1 h) resulted in a reduction of the concentration of antioxidants in surface layers due to the occurrence of oxidation process, whereas the concentrations of these compounds in the deeper tissues remained unchanged. In contrast, prolonged exposure of the fruits to ozone gas (3 h) evoked a stronger system response and probably contributed to the transport of antioxidants from internal tissues to the region of oxidative stress.

The results of our study are consistent with those presented by Glowacz et al. [17]. The TPC was increased in red bell peppers exposed to ozone at 0.1 μmol/mol concentration, while TPC was not affected when these fruits were exposed to higher concentrations of ozone (0.3 μmol/mol). Another work by the same author [18] established that there were no significant differences in terms of TPC in chili peppers. Regardless of ozone concentration, AA was not affected in red chili peppers, but was found to be significantly reduced after 14 days in green chili peppers exposed to ozone at 2 μmol/mol concentration when compared with control samples. The authors suggested that green peppers were more sensitive to ozone treatment. However, it is difficult to compare the results of these studies because in that experiment red and green chili peppers were continuously exposed to very low concentrations of ozone during the storage period at 10 °C for 14 days. In our previous study [19], the highest TPC and antiradical activity were noted in ozone-treated fruits stored for 20 days. On the basis of literature data [17,19] and obtained results, it may be concluded that during the storage period of 20 days, physiological accumulation of secondary metabolites takes place. Storage of fruits for a longer period of time results in degradation of these compounds, and the degraded products serve the role of antioxidants in the cell environment. For this reason, the highest TPC and antiradical activity for control samples was noticed at T20, which rapidly decreased during the next 10 days (Figure 1 and Figure 2). TPC has previously been found to be increased in other ozone-treated fruits and vegetables like bananas and pineapples [28], and papayas [29]. In the study of Ong et al. [29], a significant increase in the TPC of papaya fruits from 4 to 10 days of storage has been noticed. Additionally, antioxidative activity measured by the DPPH and FRAP assays also increased. Rodoni et al. [30] observed changes in these parameters during the storage of ozonated tomato fruit at a temperature of 20 °C. They noted a 50% increase in the accumulation of total phenolic compounds compared to the control after six days, and a decrease after nine days. However, in this study, no changes in the AA of fruits after ozone treatment were observed. In another experiment [31], the highest antioxidant capacity was found in the methanol extract obtained from ozonated berries. Additionally, ozone-treated raspberries showed a statistically significantly higher level of phenolic compounds and also demonstrated higher total antioxidant capacity as compared to untreated fruits [32]. The impact of ozone on health-promoting, microbiological, and color properties of *Rubus ideaus* raspberries has also been investigated. More and more attempts are being made to improve the biosynthesis of phenolic compounds and AA of fruits during pre- and postharvest stages [33,34,35].

Despite many studies, the mechanism behind the increasing concentration of phenolic compounds following ozonation process is not clear. Two probable hypotheses are proposed. One theory states that ozone can lead to the release of some of the bound phenolic compounds through partial demolition of the cell structure [36]. During exposure to ozone gas, modification of the cell wall may occur, which in turn can lead to an increase in the phenolic content of the fruits. These modifications may increase the extraction efficiency and contribute to the release of some conjugated phenolic compounds in the cell wall [28]. In addition, phenolic compounds, depending on their structure, exhibit different tendencies to accumulate within the cell wall [37]. The second theory attributes the increase to the changes in enzymes activities induced by ozone. The rapid accumulation of phenolics can be caused by the activation of preexisting enzymes [30]. This mechanism has also been confirmed by our previous research on pepper fruits [19]. We observed higher activity of guaiacol oxidase and polyphenol oxidase enzymes after ozone treatment.

The results of another study [1,11] demonstrate that ozone treatments with concentrations up to 1 μmol/mol did not show any influence on the content of phenolic compounds and antioxidant activities. Moreover, completely different results have been published by de Souza et al. [38]. A color reduction was observed in sugarcane juice by removal of phenolics. However, this was the goal of the work and, hence, high ozone concentration (3.82 mg/min) and long processing time (4 h) were used.

### 3.2. HPLC

**Figure 3:**
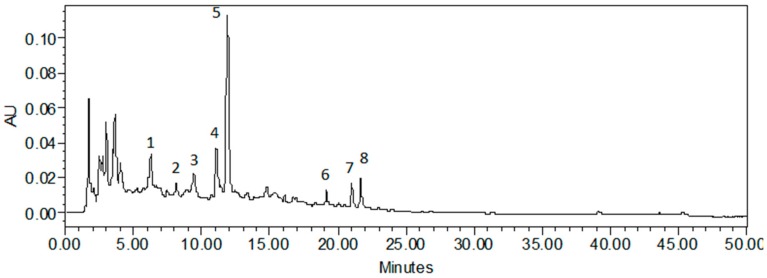
Chromatogram of the 70% aqueous methanol fraction extracted from pericarp of control pepper fruit.

**Figure 4:**
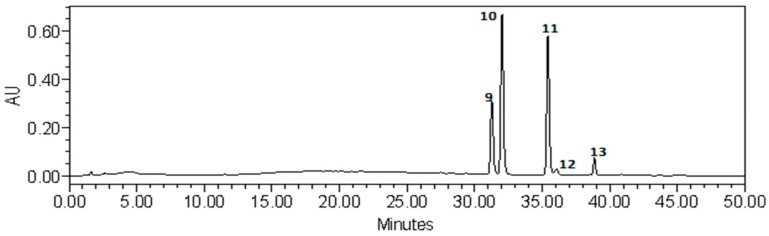
Chromatogram of the 70% aqueous methanol fraction extracted from placenta of control pepper fruit.

Chromatographic analysis of the lipophilic fraction obtained from the pericarp and placenta of pepper fruits clearly showed their different chemical compositions (Figure 3 and Figure 4). In the extracts of the pericarp, eight compounds were found, whose retention times were in the range of 5–25 min (Figure 3). In the placental extracts, the presence of other compounds was noticed, whose retention times were between 30 and 40 min (Figure 4). The obtained results confirm the fact that different anatomical parts of pepper fruits contain different compounds, despite their immediate vicinity. Similarly, in the fraction that showed medium lipophilicity, qualitative and quantitative differences were noted between the pericarp and placental extracts obtained from several pepper varieties [19,21].

For a quantitative HPLC analysis, the standard curve of quercetin 3-*O*-rhamnoside was used, since the same compound was used for the quantitative estimation of unidentified components present in 40% fraction [19]. The effect of ozonization on the content of secondary metabolites in the pericarp and placenta of pepper fruits was investigated in this study, and the results showed a slight increase in the level of these compounds when subjected to ozone treatment for 1 h, while ozonation for 3 h resulted in the accumulation of the analyzed compounds, whose concentration was almost doubled in pericarp and six-fold in placenta of pepper fruits (Table 1). The results obtained by chromatographic analysis were consistent with those obtained by spectrophotometric methods (Figure 1, Table 1). Treatment of pericarps with ozone for 1 h caused a reduction in the contents of these compounds after 20 days of ozonation (T20), while their concentration was almost doubled in pericarps treated for 3 h. It is evident from the above findings that the lower dose of ozone probably causes the degradation of antioxidants under the influence of oxidative stress, while higher intensity of the stress factor stimulates the plant to synthesize greater amounts of compounds that act as substrates for the synthesis of antioxidants. This outcome is particularly evident in placental extracts ozonated for 3 h, where the concentration of test compounds was more than five-fold higher in comparison with the control fruits on 10th day after ozonation (Table 1). To date, there have been no studies in the literature that have reported about changes in the concentration of lipophilic compounds following ozone treatment and the effect of storage on the content of these compounds in pepper fruits. In our earlier investigations, we noticed a positive effect of ozone on the level of quercetin derivatives 20 days after ozonation [19]. In the presented study, it was found that long-term storage of pepper fruits (up to 30 days from harvesting and ozonation) resulted in quantitative changes in the secondary metabolites; however, after the 20th day, the accumulation of these compounds was found to be the highest both in pericarp and placenta of pepper fruit (Table 1).

### 3.3. PCA and Correlation Analysis

**Figure 5:**
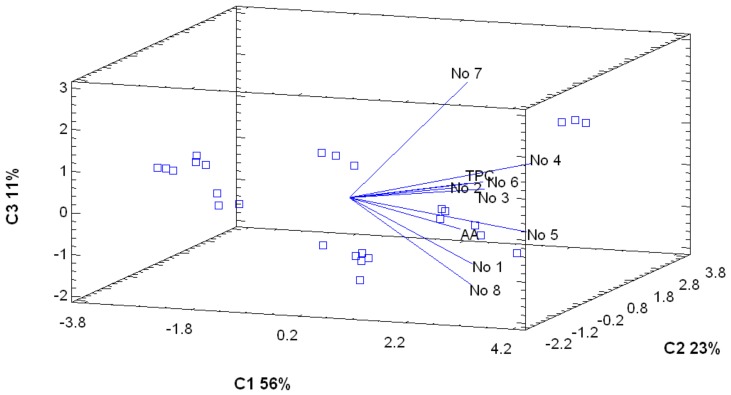
Principal components analysis of the tested parameters in pericarp. C1: Component 1; C2: Component 2; C3: Component 3; AA: Antiradical activity; TPC: Total phenolic content.

**Figure 6:**
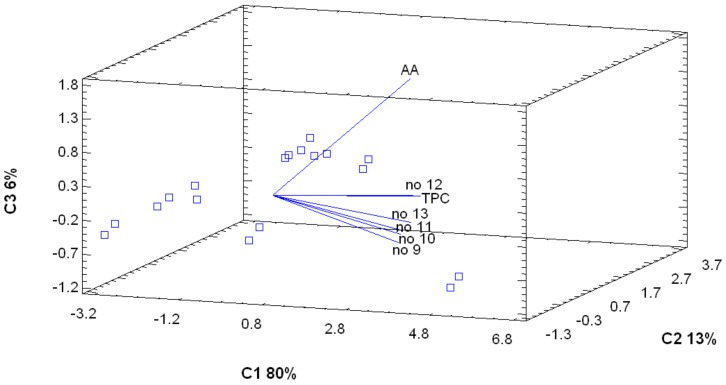
Principal components analysis of the tested parameters in placenta.

The PCA showed the presence of numerous dependencies. As shown in Figure 5, the line representing compound no. **5** is the closest to the line indicating AA. Compounds (groups) have to be isolated, identified, and checked for AA. Moreover, compound no. **7** can be suspected to have a slightly different structure than the other compounds and seems to possess less antiradical activity. While in placenta (Figure 6), the presence of DPPH value line at a quite distant position indicates that phenolic compounds present in the placental region do not show high AA.

The Pearson’s correlation analysis of the ozonation time and other tested parameters indicates numerous correlations (Table 2). The ozonation process particularly affected the placental region after 10 days of storage (T10). All the tested parameters were positively correlated with the duration of ozone treatment. Interestingly, after 20 days of storage, correlations were found to be reversed, and after 30 days (T30) they were again positive. The results for the pericarp region were slightly different. Some of the compounds showed high positive correlation with the duration of the ozonation process after 10 days of storage, while some showed after 10 days (at T20) and others after 30 days. A positive correlation was observed only for compound 7 in all the examined time periods. There was a negative linear relationship between ozonation and compound 3 at T30. The favorable influence of ozonation and antioxidative activity was only observed at T10 and T30, but not at T20. Thus, it can be concluded that ozonation causes immediate effects which seem to be sustained for a longer period of time.

## 4. Conclusions

Exposure of fruits to higher doses of ozone (3 h) induces the elicitation effect and increases the AA of the aqueous methanolic (70%) fraction of pepper extract. The concentration and activity of secondary metabolites were found to be variable during storage, with the highest values recorded on the 20th day after harvest, both in control and ozonated fruits, regardless of the ozone dosage used. Ozonation for a period of 3 h, but not 1 h, showed a positive effect on the phenolic composition and antioxidant activity during prolonged storage of pepper fruits. Three hours of ozonation seems to be the appropriate exposure time to increase the persistence of pepper fruits during storage.

## Figures and Tables

**Table 1:** Content of phenolic compounds (quercetin-3-*O*-rhamnoside equivalent mg/100 FM) in pericarp and placenta of pepper fruit as affected by ozonation and storage.

	Peak no. ^1^	Retention Time (min)	T10			T20			T30		
			Ozonation			Ozonation			Ozonation		
			0	1 h	3 h	0	1 h	3 h	0	1 h	3 h
Pericarp	1	6.2	0.527 ^c^* ± 0.010	0.425 ^d^ ± 0.009	0.709 ^ab^ ± 0.053	0.640 ^b^ ± 0.16	0.650 ^ab^ ± 0.011	0.704 ^ab^ ± 0.011	0.785 ^a^ ± 0.024	0.538 ^c^ ± 0.015	0.716 ^a^ ± 0.067
	2	8.1	0.276 ^c^ ± 0.012	0.602 ^b^ ± 0.054	0.775 ^a^ ± 0.069	0.280 ^c^ ± 0.013	0.229 ^c^ ± 0.024	0.581 ^b^ ± 0.006	0.245 ^c^ ± 0.019	0.132 ^d^ ± 0.003	0.206 ^c^ ± 0.016
	3	9.4	0.384 ^d^ ± 0.015	0.273 ^e^ ± 0.030	0.520 ^c^ ± 0.008	0.786 ^a^ ± 0.021	0.520 ^c^ ± 0.013	0.874 ^a^ ± 0.010	0.709 ^ab^ ± 0.060	0.597 ^c^ ± 0.039	0.569 ^c^ ± 0.033
	4	11.1	0.411 ^de^ ± 0.001	0.464 ^de^ ± 0.014	1.071 ^b^ ± 0.046	1.107 ^b^ ± 0.070	0.840 ^bc^ ± 0.045	1.548 ^a^ ± 0.012	0.944 ^b^ ± 0.066	0.475 ^d^ ± 0.017	0.648 ^d^ ± 0.018
	5	11.9	1.269 ^c^ ± 0.089	1.370 ^bc^ ± 0.005	3.374 ^a^ ± 0.084	3.360 ^a^ ± 0.082	1.943 ^b^ ± 0.108	3.130 ^a^ ± 0.241	2.455 ^b^ ± 0.187	1.625 ^bc^ ± 0.073	2.196 ^b^ ± 0.089
	6	19.5	0.177 ^c^ ± 0.010	0.312 ^b^ ± 0.009	0.478 ^a^ ± 0.015	0.273 ^b^ ± 0.009	0.262 ^b^ ± 0.018	0.330 ^b^ ± 0.022	0.157 ^c^ ± 0.006	0.059 ^d^ ± 0.003	0.163 ^c^ ± 0.014
	7	21,0	0.305 ^c^ ± 0.021	0.257 ^c^ ± 0.022	0.562 ^c^ ± 0.024	0.611 ^c^ ± 0.044	2.375 ^b^ ± 0.104	4.313 ^a^ ± 0.050	0.257 ^c^ ± 0.021	0.343 ^c^ ± 0.003	0.409 ^c^ ± 0.038
	8	21.7	0.170 ^d^ ± 0.011	0.225 ^d^ ± 0.023	0.571 ^ab^ ± 0.039	0.496 ^b^ ± 0.022	0.404 ^c^ ± 0.024	0.592 ^a^ ± 0.010	0.563 ^ab^ ± 0.014	0.407 ^c^ ± 0.027	0.654 ^a^ ± 0.036
	Summary		3.519	3.928	8.06	7.553	7.223	12.072	6.115	4.176	5.561
Placenta	9	31.2	0.529 ^c^ ± 0.03	0.633 ^bc^ ± 0.100	3.506 ^a^ ± 0.267	1.511 ^b^ ± 0.079	1.253 ^b^ ± 0.052	1.099 ^b^ ± 0.006	0.444 ^c^ ± 0.038	0.236 ^c^ ± 0.013	1.240 ^b^ ± 0.034
	10	31.9	1.571 ^c^ ± 0.102	2.101 ^bc^ ± 0.052	7.536 ^a^ ± 0.044	3.803 ^b^ ± 0.059	2.751 ^b^ ± 0.083	3.057 ^b^ ± 0.079	1.107 ^c^ ± 0.095	0.627 ^c^ ± 0.049	3.211 ^b^ ± 0.100
	11	35.4	1.003 ^c^ ± 0.079	1.660 ^c^ ± 0.075	6.500 ^a^ ± 0.104	3.400 ^b^ ± 0.093	2.116 ^bc^ ± 0.113	2.621 ^b^ ± 0.202	0.750 ^cd^ ± 0.014	0.547 ^cd^ ± 0.041	2.779 ^b^ ± 0.101
	12	36.0	0.074 ^cd^ ± 0.010	0.122 ^c^ ± 0.019	0.435 ^a^ ± 0.014	0.314 ^b^ ± 0.028	0.176 ^c^ ± 0.013	0.265 ^b^ ± 0.015	0.068 ^cd^ ± 0.007	0.033 ^d^ ± 0.005	0.186 ^c^ ± 0.017
	13	38.8	0.131 ^c^ ± 0.006	0.169 ^c^ ± 0.007	0.718 ^a^ ± 0.008	0.375 ^b^ ± 0.020	0.287 ^bc^ ± 0.015	0.394 ^b^ ± 0.020	0.122 ^c^ ± 0.009	0.066 ^c^ ± 0.009	0.279 ^bc^ ± 0.026
	Summary		3.308	4.685	18.695	9.403	6.583	7.436	2.491	1.509	7.695

Notes: ^1^ Peak numbers are the same as given in Figure 1 and Figure 2. * The data are expressed as the mean (*n* = 3) ± SD; for each peak, values not sharing the same letter within the same row were significantly different at *p* < 0.05.

**Table 2:** Pearson’s correlation analysis of ozonation time and other tested parameters.

Parameter	T10	T20	T30
Pericarp			
1	0.6308	**0.9255**	−0.2723
2	**0.9848**	0.7904	−0.3420
3	0.5498	0.2396	**−0.9449**
4	0.8997	0.6167	−0.6241
5	0.8864	−0.1512	−0.3046
6	**0.9983**	0.7785	0.0585
7	0.7826	**0.9996**	**0.9973**
8	**0.9221**	0.5141	0.3656
HPLC sum	**0.9037**	**0.8339**	−0.2772
%AA	0.8009	0.1132	0.7551
TPC	0.8094	−0.0173	**0.9556**
Placenta			
9	**0.9002**	**−0.9895**	0.7508
10	**0.9034**	−0.6890	0.7653
11	**0.9155**	−0.6026	**0.8219**
12	**0.9209**	−0.3485	0.7375
13	**0.8932**	0.1704	0.7131
HPLC sum	**0.9072**	−0.6799	0.7827
%AA	**0.9463**	−0.5770	0.7580
TPC	**0.9373**	−0.5906	−0.5051

Notes: Values in bold are statistically significant at *p* < 0.05.
